# An associative model of Chinese university teachers' personality traits and students' classroom engagement from the teacher's perspective: the mediating role of emotional regulation

**DOI:** 10.3389/fpsyg.2026.1823628

**Published:** 2026-06-09

**Authors:** Baohong Lu, Wenzhi Hou, Jinliang Sun, Lijun Wang

**Affiliations:** 1 Department of Inclusive Education, Shinhan University, Gyeonggi-do, Uijeongbu, Republic of Korea; 2 Institute of Physical Education, Xingyi Normal University for Nationalities, Guizhou, Xingyi, China; 3 Department of Public Physical, Changchun Humanities and Sciences College, Jilin, Changchun, China; 4 Department of Basic Courses, Suzhou City University, Jiangsu, Suzhou, China; 5 International College, Krirk University, Bangkok, Thailand

**Keywords:** big five personality traits, classroom engagement, emotional regulation, structural equation modeling (SEM), teacher's perspective

## Abstract

**Background:**

Teachers' personality traits and emotional regulation are pivotal in shaping the instructional climate, yet the specific mechanisms through which these factors are collectively associated with student engagement from the teacher's perspective remain under-explored, particularly within a unified structural framework.

**Objective:**

This study aimed to construct and validate a structural associative model to explore the association of Chinese university teachers' Big Five personality traits with students' classroom engagement.

**Methods:**

A convenience sample of 154 university teachers from diverse regions and academic disciplines across China completed the NEO-PI-R (Chinese version), the Emotion Regulation Questionnaire, and an adapted Classroom Engagement Scale. A second-order structural equation model (SEM) with bootstrapping (5,000 resamples) was conducted to test the hypothesized mediation pathways.

**Results:**

The second-order SEM exhibited an excellent fit to the data (χ^2^/df = 1.636, CFI = 0.940, TLI = 0.930, RMSEA = 0.064). Findings revealed that teacher personality, as a holistic construct, was significantly and positively associated with students' classroom engagemen (β = 0.636, *p* < 0.001). Notably, the Openness dimension showed a robust factor loading (β = 0.919), affirming the theoretical integrity of the model. Furthermore, emotional regulation significantly mediated the link between personality traits and engagement (indirect effect = 0.255, *p* < 0.001), accounting for 32.1% of the total effect.

**Conclusion:**

The results underscore that while teachers' stable dispositions provide a foundation for student involvement, adaptive emotional regulation—specifically cognitive reappraisal—may serve as a bridge linking these traits to engagement outcomes. This study provides empirical evidence for integrating emotion-management training into professional development to optimize teacher-student interactions in higher education.

## 1 Introduction

In recent years, higher education in China experienced significant development, with the level of educational accessibility continuing to improve. As the gross enrollment rate in higher education rapidly increased, teaching quality and student learning outcomes became focal points of attention. Despite the notable improvement in educational resources and technological capabilities, there remained considerable room for enhancing students' classroom engagement. As a core dimension of student learning activities, classroom engagement not only reflected students' enthusiasm and concentration in the learning process but also played a crucial role in improving learning outcomes, critical thinking, and overall competence (Sökmen et al., 2024). Studies showed that students with higher levels of classroom engagement had advantages in academic achievement and social skill development. However, in many university classrooms, students' behavioral, cognitive, and emotional engagement remained insufficient, which was closely related to teachers' teaching behavior, classroom management styles, and the quality of teacher-student interactions (Dang et al., 2025). Thus, exploring key factors influencing students' classroom engagement held significant theoretical and practical value for improving educational quality.

In classroom teaching, teachers not only served as knowledge transmitters but also as shapers of the classroom atmosphere and motivators of student learning. Teachers' personality traits played an important role in influencing teaching behavior and classroom outcomes (Izydorczyk et al., 2025). According to the Big Five Personality Theory, teachers with different personality traits exhibited significant differences in teaching styles, classroom management approaches, and teacher-student interaction patterns, which could have profound effects on students' classroom engagement (Qu and Wang, 2024). For instance, teachers high in openness tended to adopt innovative teaching methods that captured students' attention, while teachers high in conscientiousness were more likely to focus on structured classroom management and achieving instructional goals (Holl et al., 2024). These behaviors significantly **shape** students' participation in the classroom. Therefore, investigating the relationship between teachers' personality traits and students' classroom engagement is critical to understanding the **predictive mechanisms** underlying the enhancement of teaching quality. Beyond teachers' personality traits, emotional regulation, as a psychological mechanism, potentially served as a mediator between teachers' personality traits and students' classroom engagement (Ilana, 2024). Emotional regulation theory suggests that teachers' ability to manage emotions during the teaching process shapes the quality of their interactions with students and the harmony of the classroom atmosphere (Zitzmann et al., 2024). For example, teachers who effectively regulated their emotions were more likely to maintain a positive teaching state under stressful conditions, thereby creating a more engaging classroom environment. Emotional regulation not only alleviated the interference of negative emotions in teaching behavior but also enhanced teachers' confidence and control in classroom management, which collectively impacted students' classroom engagement (Shdaifat et al., 2024). Thus, emotional regulation represents a potential pathway through which teachers' personality traits shape students' classroom engagement.

This study goes beyond simple correlation analysis and aims to construct and validate an integrative “trait-emotion-participation” structured associative model from the perspective of teacher observation. The model treats university teachers' personality traits as a second-order latent variable, systematically exploring how they are related to students' behavioral, cognitive, and emotional engagement. Building on this, the study provides an in-depth analysis of the mediating role of emotional regulation in this process and the strength of its indirect effects, thereby revealing how stable personality traits may be linked to student classroom participation through dynamic emotional management strategies. Finally, the structural equation modeling (SEM) is employed to validate the model's empirical fit within the unique context of China's higher education, offering scientific theoretical support and practical insights for optimizing teacher-student interaction mechanisms (Stulcbauer et al., 2024).

The **primary overarching objective** of this study is **to construct a unified structural framework** that reveals the “trait–emotion–engagement” mechanism. By employing SEM, we seek to validate how teachers' stable dispositions are translated into perceived student engagement via adaptive emotional strategies. By constructing and validating this predictive mechanism model from the teacher's perspective, this study provides theoretical insights and practical references for optimizing teachers' instructional behaviors and enhancing students' learning outcomes. This research contributed to filling the current theoretical gap regarding the relationship between teachers' personality traits and students' classroom engagement. Existing studies predominantly focused on factors such as classroom teaching methods and students' learning strategies, while lacking systematic exploration of the deeper connections between teachers' individual traits and student learning interactions (Qu and Wang, 2024). By incorporating the Big Five Personality Theory and emotional regulation theory, this study constructed a multidimensional theoretical framework to examine how teachers' personality traits predict students' classroom engagement from the teacher's perspective, offering a new perspective for enriching the theoretical system of educational psychology and pedagogy. The research also provided targeted guidance for professional development and classroom teaching improvement among university teachers. By uncovering the strengths and weaknesses of teachers with different personality traits in classroom management and instructional behaviors, this study offered a scientific basis for designing teacher training programs. Furthermore, by highlighting the critical role of emotional regulation in the teaching process, this research could help teachers enhance their emotional management abilities to better address complex challenges in teaching contexts. Ultimately, such practical guidance contributed to improving the quality of university classroom teaching and promoting comprehensive improvements in students' learning outcomes.

Despite numerous studies examining the effects of teachers' personality traits (e.g., Extraversion, Conscientiousness) on students' classroom engagement and the role of emotional regulation in teaching contexts (Izydorczyk et al., 2025; Qu and Wang, 2024; Holl et al., 2024; Ilana, 2024; Zitzmann et al., 2024; Shdaifat et al., 2024), two gaps remain:

Prior research has often focused on the direct relationship between a single personality dimension and classroom engagement, without systematically assessing the joint mechanisms and interactions of all five personality factors. To address this, our study is the first to employ the full NEO-PI-R five-factor framework to construct a “Teacher Personality–Emotional Regulation–Student Engagement” triadic interaction model, thereby revealing the distinct pathways and strengths of each dimension's effect on classroom engagement.

Although some studies have suggested that emotional regulation may serve as a mediator of teaching effectiveness, this research has largely been confined to primary or secondary school settings, with a lack of empirical validation in higher education. Our study innovatively introduces Gross et al.'s emotional regulation theory into the university teaching context and employs a bootstrapping mediation analysis, filling the empirical gap concerning emotional regulation as a mediator in the college classroom environment.

A review of existing literature indicates that, although studies have separately demonstrated the direct impact of Extraversion, Conscientiousness, and other Big Five dimensions on classroom engagement and the role of emotional regulation in teacher–student interactions, three major gaps remain:

Most research focuses on a single personality dimension, lacking systematic comparisons across the full five-factor framework;

Emotional regulation process models have predominantly been applied in primary or secondary school contexts, with no empirical validation in higher education settings;

While sporadic studies suggest that emotional regulation may mediate the link between personality and teaching outcomes, this mediating mechanism and overall model fit have not been examined concurrently within a unified SEM framework.

To address these gaps, this study integrates NEO-PI-R's five-factor theory and Gross et al.'s emotional regulation framework to construct a “Personality Traits → Emotional Regulation → Classroom Engagement” model from the teacher's perspective. On one hand, we simultaneously assess the relative effects of all five dimensions within a single sample; on the other, we employ bootstrapping mediation analysis and SEM fit-index evaluation to reveal the unique mediating role of emotional regulation. Accordingly, we propose the following hypotheses:

Hypothesis 1a: teachers' extraversion, agreeableness, and conscientiousness positively predict students' behavioral, cognitive, and emotional engagement.

Hypothesis 1b: teachers' neuroticism negatively predicts students' classroom engagement.

Hypothesis 1c: teachers' openness positively predicts students' classroom engagement.

Hypothesis 2: emotional regulation mediates the relationship between teachers' personality traits (the five factors) and students' classroom engagement.

Unlike prior research that treats personality dimensions as isolated predictors, this study argues that the teacher's psychological makeup functions as a synergistic system. Examining a single trait (e.g., Extraversion) in a vacuum risks oversimplifying the instructional climate, as the positive impact of sociability may be moderated or nullified by high levels of Neuroticism or low Conscientiousness. By integrating the full Five-Factor framework, we move beyond incremental “variable-adding” and instead offer a holistic mapping of how stable teacher dispositions collectively drive student engagement through the specific situational catalyst of emotional regulation.

## 2 Literature review

### 2.1 Theoretical basis of teachers' personality traits

A growing body of research has established that individual personality traits shape classroom engagement: for example, teachers' Extraversion and Conscientiousness are consistently associated with greater student participation, while high Neuroticism can undermine the classroom climate (Shdaifat et al., 2024; Maksimović et al., 2024; Wang et al., 2024; Paula et al., 2024; Zadok et al., 2024; Isbulan et al., 2024; Hasani et al., 2024; Ciydem et al., 2023; Eggermont et al., 2023; Sywulka, 2024). According to the NEO-PI-R framework (Paula et al., 2024), these traits encompass five distinct dimensions: neuroticism (emotional instability), Extraversion (sociability and energy), Openness to Experience (intellectual curiosity and creativity), Agreeableness (altruism and trust), and Conscientiousness (self-discipline and goal-orientation). However, much of this work remains fragmented—examining one or two traits in isolation—and yields mixed results depending on context and measurement (Maksimović et al., 2024; Zadok et al., 2024). Moreover, the reliance on predominantly lower-impact outlets and the near-exclusive focus on primary and secondary education settings limits the generalizability to higher education. A more systematic synthesis across all five factors is therefore needed to clarify which traits exert the most robust effects on the multidimensional construct of student engagement: behavioral (effort and persistence), cognitive (mental investment and self-regulation), and emotional (affective reactions and interest) engagement (Sywulka, 2024; Briffett-Aktaş and Ying, 2025; Lin et al., 2025; Du et al., 2025; Oseghale et al., 2025; Oruç, 2024; Arwa, 2024; Aldrup et al., 2024).

Although several influential recent studies have examined teacher-student interactions, most employed “black-box” approaches that directly linked teaching styles to instructional outcomes without considering the underlying personality traits shaping these styles. For instance, while some research emphasizes the “orchestration” mechanism of teaching engagement, our study advances this theory by positioning teachers' personality traits as the primary orchestrators and demonstrating that their instructional actions are achieved through strategic emotional regulation. This pivotal perspective not only explains the mechanisms underlying teaching engagement but also reveals why specific types of teachers are more effective at fostering engagement in high-stakes environments such as university classrooms.

Emotional regulation has been proposed as a key mechanism through which teachers translate their dispositional tendencies into interactive classroom behaviors (Hsu et al., 2024; Lemay et al., 2024; Zhu, 2024; Sun and Yin, 2024; Namaziandost and Rezai, 2024; Mueller et al., 2024). According to Gross's process model (Zhu, 2024), teachers primarily deploy two distinct strategies: cognitive reappraisal, a proactive method involving the reinterpretation of stressful situations to alter their emotional impact, and expressive suppression, a reactive strategy aimed at inhibiting ongoing emotion-expressive behavior. These strategies enable teachers to maintain positive affect and instructional clarity under stress, thereby fostering an open and supportive learning environment. Yet empirical tests of this mediating role remain scarce in real-world university settings. Existing studies often rely on self-report data from younger student populations, neglect *in-situ* observations of teacher–student interactions and the dynamic emotional demands of higher education classrooms.

Critically, few studies have integrated personality and emotional regulation within a unified structural framework, leaving the relative contributions and interactive pathways of these constructs unexplored. The present research addresses this gap by employing SEM to model how each Big Five dimension predicts engagement directly and indirectly via emotional regulation from the teacher's perspective—offering both theoretical integration and empirical rigor beyond the piecemeal approaches of prior work.

### 2.2 Related research on students' classroom engagement

Classroom engagement—a multidimensional construct comprising behavioral, cognitive, and emotional facets—has long been heralded as a cornerstone of effective learning (Sywulka, 2024; Briffett-Aktaş and Ying, 2025; Lin et al., 2025; Du et al., 2025; Oseghale et al., 2025; Oruç, 2024). Behavioral engagement refers to the extent of students' active involvement in learning tasks, encompassing observable indicators such as effort, persistence, and task completion (Sywulka, 2024; Briffett-Aktaş and Ying, 2025). Cognitive engagement involves students' psychological investment in learning, characterized by the use of self-regulatory strategies and deep processing of content through problem analysis (Lin et al., 2025). Emotional engagement reflects students' affective reactions within the classroom, including their interest, positive attitudes, and sense of belonging (Du et al., 2025).

Critically, these dimensions rarely operate in isolation: a supportive classroom atmosphere and interactive pedagogy can simultaneously foster students' willingness to act, think deeply, and feel connected (Hu et al., 2024). However, much research remains descriptive—cataloging correlations between teacher behaviors and individual engagement facets—without unpacking the dynamic interplay among teaching methods, emotional climate, and student motivation (Arwa, 2024; Aldrup et al., 2024). Moreover, studies often draw from limited contexts and lower-impact outlets, leaving questions about measurement validity and theoretical coherence unanswered.

This fragmented landscape underscores the need to integrate emotional regulation as a central mechanism. According to Gross's process model (Hsu et al., 2024), teachers' ability to manage stress—primarily through cognitive reappraisal (proactively reinterpreting events) or expressive suppression (inhibiting emotional displays)—shapes not only the affective tone of the classroom but also students' willingness to engage behaviorally and cognitively under challenge. Yet empirical tests of this mediating role, particularly in authentic higher-education settings, are virtually absent. By embedding these specific emotional regulation strategies within a unified SEM framework alongside the multidimensional engagement measures, the present study seeks to move beyond piecemeal description toward a cohesive predictive model that reflects the complexity of real-world teaching and learning.

### 2.3 The mechanism of emotional regulation

Emotional regulation—defined as the process through which individuals manage and modulate their emotional experiences, including which emotions they have, when they occur, and how they are expressed—has been identified as a critical skill in educational settings, linking teacher disposition to student engagement and learning outcomes (Hsu et al., 2024). Foundational theories, such as Gross's process model, suggest that teachers primarily navigate emotional demands through two distinct strategies: cognitive reappraisal, a proactive antecedent-focused strategy involving the cognitive reinterpretation of a situation to alter its emotional impact, and expressive suppression, a reactive response-focused strategy aimed at inhibiting the outward expression of ongoing emotions (Sun and Yin, 2024). While theories like Ellis's ABC Model highlight how beliefs mediate emotional responses (Zhu, 2024), teachers who predominantly deploy adaptive strategies like reappraisal can transform potentially disruptive feelings into opportunities for constructive interaction. However, existing empirical work remains largely descriptive and confined to K−12 contexts, often neglecting the dynamic emotional demands of higher education (Namaziandost and Rezai, 2024; Mueller et al., 2024).

Critically, scant research has tested emotional regulation's putative mediating role within a comprehensive model that simultaneously accounts for the full Big Five personality framework—comprising Neuroticism, Extraversion, Openness, Agreeableness, and Conscientiousness—and robust engagement outcomes. Without such integration, the field risks perpetuating fragmented insights rather than revealing how teachers' trait-driven emotional management concretely shapes classroom climate and downstream engagement behaviors. The present study addresses this gap by embedding these dual emotional regulation strategies within a structural equation model alongside all five personality dimensions and multi-facet engagement measures, thereby offering a theoretically coherent test of emotional regulation as the linchpin converting teacher personality into enhanced student engagement.

### 2.4 Research hypotheses

Hypothesis 1 (H1): teachers' personality traits, as a holistic construct, significantly and positively predict students' multidimensional classroom engagement.

Hypothesis 2: emotional regulation mediated the relationship between teachers' personality traits and students' classroom engagement.

## 3 Research methods

### 3.1 Research design

This study utilized a cross-sectional, correlational survey design. Data were collected from a convenience sample of university teachers from diverse regions and academic disciplines across China to examine the associations among personality traits, emotional regulation strategies, and perceptions of student classroom engagement. It is critical to note that the subsequent structural equation modeling (SEM) was applied to test the hypothesized mediation pathways and associative relationships; therefore, terms such as “predict” or “effect” denote statistical associations within the model and do not imply causal inferences.

### 3.2 Sample and data collection

To ensure the diversity and quality of the research data, this study utilized the “Questionnaire Star” platform to recruit participants across various regions and disciplines in China. The data collection and cleaning process were conducted through the following four rigorous stages:

(1) Participant Recruitment: a non-probability convenience sampling approach was employed. To enhance representativeness, recruitment invitations were distributed to university teachers across different geographical regions and academic disciplines, encouraging participation from a broad professional spectrum.
(2) Informed Consent: a unique invitation link was distributed to each potential participant to prevent duplicate responses. All participants were fully informed of the study's anonymous nature and provided electronic informed consent before proceeding.
(3) Data Quality Control: to ensure data integrity, the questionnaire included three “attention check” items (e.g., “Please select “Strongly Agree” for this item”). Only questionnaires completed within a reasonable duration (300–900 s) were retained, filtering out responses with irregular or random patterns.
(4) Final Sample Selection: from the initially collected responses, 154 valid cases were ultimately retained after rigorous screening (a valid recovery rate of 85.6%). While the sample size is relatively small, the case-to-parameter ratio met the exploratory requirements for maximum likelihood estimation in structural equation modeling (SEM). Detailed demographic information of the participants is presented in Table 1.

**Table 1:** Demographic Characteristics of the Participants (*N* = 154).

Variable	Category	Frequency (*n*)	Percentage (%)
Gender	Male	58	37.66
	Female	96	62.34
Subject	Education	43	27.92
	Public administration	33	21.43
	Chinese/Literature	29	18.83
	History/Philosophy	25	16.23
	Statistics/Computer Science	24	15.59
Region	North China	31	20.13
	South China	31	20.13
	Central China	21	13.64
	Western China	33	21.43
	Eastern China	38	24.67
Age	Under 30 years old	113	73.38
	31–40 years old	26	16.88
	41–50 years old	13	8.44
	51 years old and above	2	1.3
(Teaching Exp.)	0–5 years	77	50
	6–10 years	43	27.92
	11–15 years	19	12.34
	16–20 years	11	7.14
	21 years and above	4	2.6
Professional title	Lecturer	114	74.03
	Associate Professor	12	7.79
	Professor	28	18.18
Educational background	Bachelor's Degree	91	59.09
	Master's Degree	29	18.83
	Doctoral Degree (PhD)	34	22.08

To further justify the sample size adequacy, we conducted a *post hoc* power analysis using the semPower R package (version 2.2.0, R Foundation for Statistical Computing, Vienna, Austria). Assuming a target power of 0.80, an alpha level of 0.05, and a conservative model with 20 degrees of freedom, a sample of 154 is sufficient to detect a medium effect (RMSEA = 0.07) but may be underpowered for smaller effects. Readers should interpret the null findings or small indirect effects with caution.

Regarding sampling limitations, the use of convenience sampling via an online platform (Wenjuanxing) may have introduced self-selection bias. Teachers who voluntarily participated might differ systematically from non-participants (e.g., in their interest in teaching quality, emotional awareness, or digital literacy). Consequently, the sample may not be fully representative of all Chinese university teachers. Future research should consider probability-based sampling or stratified random sampling to enhance external validity.

### 3.3 Data collection process

This study focused on teachers from Chinese higher education institutions, ultimately collecting 154 valid questionnaires. Participants were recruited through convenience sampling from various universities across China to ensure a diverse representation of academic disciplines. While efforts were made to include participants from different regions, the sample size is acknowledged as exploratory in nature, with findings primarily reflective of the surveyed group. This sample size, however, satisfied the minimum case-to-parameter requirements for structural equation modeling (SEM), providing a functional basis for evaluating the hypothesized structural pathways. While efforts were made to include participants from different regions, the sample size is acknowledged as exploratory in nature, with findings primarily reflective of the surveyed group.

The research utilized well-recognized and validated instruments to ensure the reliability and validity of data collection. Specifically, (1) teachers' personality traits were measured using the Chinese version of the NEO Five-Factor Inventory (NEO-FFI), a 60-item self-report scale (or specify your actual items) utilizing a 5-point Likert scale; (2) students' classroom engagement was assessed through an adapted version of Kong's (Jusri and Lechner, 2024) Student Engagement Questionnaire, comprising 15 items across behavioral, cognitive, and emotional dimensions; and (3) emotional regulation strategies were evaluated using the Emotional Regulation Questionnaire (ERQ) developed by Gross et al. and revised by Wang et al. (2007), which measures cognitive reappraisal and expressive suppression. All scales demonstrated acceptable internal consistency within the current sample.

Data collection was conducted via the online platform Wenjuanxing. To ensure data authenticity and participant privacy, the survey was administered anonymously. Before the formal assessment, the research team provided informed consent and explained the study's purpose and confidentiality measures. Following data collection, a rigorous cleaning process—including attention checks and response-time filtering—was implemented to ensure the accuracy and credibility of the final dataset.

### 3.4 Measurement tools

#### 3.4.1 Teacher personality traits assessment tool

Teacher personality was assessed using a shortened Chinese version of the Big Five Inventory, based on the NEO framework (Xu, 2019). The scale comprises 35 items rated on a 5-point Likert scale, measuring five first-order dimensions: extraversion (seven items), agreeableness (seven items), conscientiousness (seven items), neuroticism (seven items), and openness to experience (seven items). In the current sample, the sub-scales demonstrated high internal consistency, with Cronbach's α ranging from 0.812 to 0.903, and an overall scale α of 0.893. Standardized factor loadings for all dimensions exceeded 0.47, with Openness, Conscientiousness, and Agreeableness surpassing 0.90, confirming the structural integrity and convergent validity of the Big Five construct.

#### 3.4.2 Student classroom engagement measurement tool

Student classroom engagement was measured using an adapted version of the scale developed by Kong (2003). To ensure suitability for diverse instructional contexts, references to specific subjects were replaced with “classroom activities.” A panel of three educational psychology experts reviewed the adapted items for content relevance and clarity, and a pilot test with 30 university teachers (not included in the final sample) confirmed that the revised wording was appropriate for general classroom settings. The final instrument comprised 21 items across three dimensions: behavioral engagement (seven items, reflecting effort and persistence), cognitive engagement (seven items, reflecting strategic investment), and emotional engagement (seven items, reflecting affective reactions). Responses were recorded on a 5-point Likert scale. In this study, Cronbach's α coefficients for the three dimensions were 0.85, 0.86, and 0.88, respectively. Confirmatory factor analysis (CFA) indicated a good model fit (χ^2^/df = 1.98, CFI = 0.92, RMSEA = 0.065), with composite reliability (CR) ranging from 0.87 to 0.89 and average variance extracted (AVE) from 0.52 to 0.55, ensuring robust reliability and validity.

#### 3.4.3 Emotional regulation ability assessment tool

Teachers' emotional regulation was evaluated using the Chinese version of the Emotion Regulation Questionnaire (ERQ), originally developed by Gross et al. and revised by Wang et al. (2007). Although the ERQ was initially validated in student and general adult populations, it has been successfully applied in teacher samples in prior research. The scale consists of 14 items measuring two distinct strategies: cognitive reappraisal (six items), an antecedent-focused strategy involving the reinterpretation of evocative stimuli to modify their emotional impact; and Expressive Suppression (four items), a response-focused strategy aimed at inhibiting ongoing emotion-expressive behavior. Responses were measured on a 5-point Likert scale (1 = strongly disagree to 5 = strongly agree). In the present sample, we conducted a confirmatory factor analysis (CFA) specifically for the teacher respondents, which confirmed the two-factor structure with acceptable fit (χ^2^/df = 2.10, CFI = 0.91, RMSEA = 0.079) and factor loadings above 0.70 for all items. Thus, the scale demonstrated adequate psychometric properties in this teacher sample, providing a rigorous foundation for analyzing how these dual strategies mediate the relationship between personality and engagement.

In accordance with the non-experimental correlational research design, data analysis was performed using SPSS 26.0 and AMOS 26.0 (IBM Corp., Armonk, NY, United States). Following a rigorous two-step structural equation modeling (SEM) approach, we examined the predictive pathways among the variables rather than establishing definitive causal links. The analysis proceeded as follows:

Descriptive statistical analysis was conducted to summarize teachers' demographic characteristics and the overall distribution of all research variables. Means, standard deviations, and frequency distributions provided the empirical foundation for subsequent modeling.

Common method bias (CMB) was assessed using Harman's single-factor test. The results indicated that the variance explained by the first factor was 28.7%, well below the critical threshold of 40%, suggesting that CMB did not pose a significant threat to the validity of the findings.

Measurement model assessment was performed through confirmatory factor analysis (CFA). To ensure the robustness of latent variable constructs, reliability and validity were verified using Cronbach's α for internal consistency and construct validity analysis. This step confirmed that the theoretical structures of the scales were well-represented by the observed data in the Chinese higher education context.

Structural model testing was employed to evaluate the hypothesized “Teacher Personality → Emotional Regulation → Student Engagement” mechanism. Given the exploratory nature of the study and the sample size (*N* = 154), the mediating effects were tested using the Bootstrapping method with 5,000 resamples. This non-parametric approach provided bias-corrected 95% confidence intervals (CIs) for total, direct, and indirect effects. The Bootstrapping method is particularly suited for our sample size as it offers high statistical efficiency and robust estimates for complex mediation pathways without assuming multivariate normality.

By integrating these analytical methods, this study rigorously validated the predictive relationships between the variables, offering empirical evidence to inform the enhancement of instructional practices.

## 4 Results

### 4.1 Descriptive statistics, common method bias assessment, and correlation analysis of teachers' personality traits, emotional regulation, and students' classroom engagement

To assess common method bias (CMB), we conducted Harman's single-factor test, which showed that a single factor accounted for 28.7% of the total variance—well below the 40% threshold—indicating that CMB was negligible. Next, we performed multicollinearity diagnostics on the 10 lower-order dimensions; all variance inflation factors (VIFs) were below three and tolerance values above 0.3, demonstrating no serious collinearity. Finally, using the Fornell–Larcker criterion, we confirmed that each dimension's average variance extracted (AVE) exceeded its squared inter-construct correlations, verifying good discriminant validity among the Big Five traits, the three classroom engagement dimensions, and the two emotional regulation dimensions.

To further assess discriminant validity, we calculated the heterotrait-monotrait (HTMT) ratio for all latent construct pairs. The HTMT values ranged from 0.71 to 0.88, all below the conservative threshold of 0.85 (or below 0.90 for a more lenient criterion), supporting that each construct is empirically distinct from the others. Notably, the highest HTMT value (0.88) was observed between Openness and Conscientiousness, which aligns with the conceptual overlap between these two personality dimensions. Despite this, the results confirm that multicollinearity is not a serious threat to the structural estimates, consistent with the variance inflation factors (all VIFs <3) reported earlier.

Table 2 data showed that the mean values for the dimensions of teachers' personality traits were around 4.00. Specifically, Extraversion (*M* = 4.04, SD = 0.87), Agreeableness (*M* = 4.03, SD = 0.83), and Conscientiousness (*M* = 4.03, SD = 0.80) had slightly higher scores, while Openness to Experience (*M* = 3.97, SD = 0.81) and Neuroticism (*M* = 3.40, SD = 1.10) were relatively lower. The overall mean value for personality traits was *M* = 4.02 (SD = 0.77), indicating that teachers' personality traits were generally at a high level and exhibited a relatively concentrated distribution. For students' classroom engagement, the mean value for behavioral engagement was *M* = 3.79 (SD = 0.76), for cognitive engagement *M* = 3.02 (SD = 0.59), and for emotional engagement *M* = 5.13 (SD = 1.23). The overall mean for classroom engagement was *M* = 5.21 (SD = 1.27), with emotional engagement scoring significantly higher than the other dimensions.

**Table 2:** The descriptive statistics for teachers' personality traits, emotional regulation, and students' classroom engagement.

Variables	Mean	SD	Minimum value	Maximum value
Extraversion	4.04	0.87	1	5
Agreeableness	4.03	0.83	1	5
Conscientiousness	4.03	0.80	1	5
Openness to experience	3.97	0.81	1	5
Neuroticism	3.40	1.10	1	5
Personality traits	4.02	0.77	1	5
Behavioral engagement	3.79	0.76	1	5
Cognitive engagement	3.02	0.59	0.8	4
Emotional engagement	5.13	1.23	1	7
Classroom engagement	5.21	1.27	1	7
Cognitive reappraisal	5.17	1.21	1	7
Expressive suppression	3.90	0.76	1	5
Emotional regulation	3.61	0.66	0.93	4.67

In terms of emotional regulation, the mean value for cognitive reappraisal was *M* = 5.17 (SD = 1.21), which was significantly higher than expressive suppression *M* = 3.90 (SD = 0.76). The overall mean score for emotional regulation was *M* = 3.61 (SD = 0.66), indicating that teachers demonstrated stronger abilities in cognitive reappraisal compared to expressive suppression.

Table 3 presents the descriptive statistics and correlation analysis, providing a solid empirical foundation for the subsequent mediation model. The results indicate that all five dimensions of teacher personality traits (Extraversion, Agreeableness, Conscientiousness, Openness, and Neuroticism) are significantly and positively correlated with both student classroom engagement and teachers' emotional regulation strategies, with correlation coefficients (*r*) ranging from 0.421 to 0.938 (*p* < 0.01). Specifically, Conscientiousness exhibits the strongest correlation with Behavioral Engagement (*r* = 0.876), suggesting that rigorous and responsible teacher characteristics significantly drive student involvement in the classroom. Furthermore, both emotional regulation strategies—Cognitive Reappraisal and Expressive Suppression—show strong correlations with all dimensions of classroom engagement, prefiguring their critical mediating potential in the pathway from personality traits to student engagement.

**Table 3:** Teachers “personality traits, students” classroom participation and emotional regulation relationship diagram.

Variables	Mean	SD	Extraversion	Agreeableness	Conscientiousness	Openness	Neuroticism	Behavioral engagement	Cognitive engagement	Emotional engagement	Cognitive reappraisal	Expressive suppression
Extraversion	4.043	0.868	1									
Agreeableness	4.028	0.825	0.938	1								
Conscientiousness	4.035	0.803	0.871	0.901	1							
Openness	3.974	0.806	0.831	0.881	0.896	1						
Neuroticism	3.438	1.078	0.432	0.421	0.432	0.508	1					
Behavioral engagement	4.024	0.773	0.847	0.866	0.876	0.865	0.456	1				
Cognitive engagement	3.821	0.745	0.739	0.733	0.734	0.745	0.560	0.830	1			
Emotional engagement	3.746	0.756	0.622	0.616	0.641	0.647	0.584	0.711	0.836	1		
Cognitive reappraisal	4.427	1.059	0.659	0.675	0.689	0.662	0.506	0.717	0.748	0.808	1	
Expressive suppression	36.143	8.586	0.687	0.662	0.686	0.653	0.507	0.738	0.735	0.784	0.943	1

*p* < 0.05; *p* < 0.01.

Regarding data distribution, with the exception of Neuroticism, the mean scores (*M*) for all other personality dimensions and engagement indicators are above the moderate level (*M* > 3.7), with reasonable standard deviations (SD), reflecting that the research sample possesses good representativeness and discriminative power. Notably, the significant correlation between Openness and other variables (e.g., *r* = 0.745 with Cognitive Engagement) addresses the lack of attention to this dimension in previous studies and fully supports the theoretical framework of the Big Five model. In summary, the significant correlations among the variables confirm the rationality of the research hypotheses and provide ample data support for a deeper exploration of the mediating effect of teachers' emotional regulation.

### 4.2 Model construction and validation

**Figure 1:**
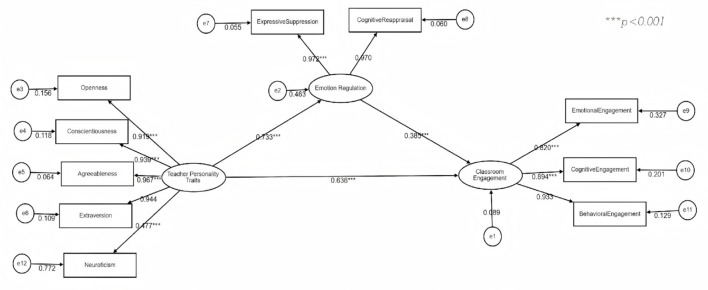
The structural mechanism model of teacher personality traits predicting students' classroom engagement from the teacher's perspective. Model Fit: χ^2^/df = 1.636, CFI = 0.940, TLI = 0.930, RMSEA = 0.064.

To deeply explore the structural associations among teachers' personality traits, emotional regulation, and students' classroom engagement. The model fit results demonstrate that all fitting indices have reached ideally recognized academic standards (χ^2^/df = 1.636, RMSEA = 0.064, CFI = 0.940, TLI = 0.930), indicating a high degree of congruence between the theoretical model and the observed data, as well as excellent statistical robustness of the model (Figure 1). At the measurement level, this study successfully constructed a second-order factor model of teachers' personality traits. The results show that all five dimensions of the Big Five personality traits exhibit significant and high-level factor loadings on their higher-order constructs, with standardized coefficients (β) ranging from 0.477 to 0.967 (*p* < 0.001). Notably, the standardized loading for the Openness dimension is as high as 0.919, providing strong support for the theoretical integrity of the personality construct. Simultaneously, as the dependent variable, the factor loadings for the three sub-dimensions of student classroom engagement (all > 0.820) also verify the effectiveness of the measurement instrument.

In terms of path analysis, teachers' personality traits exert a potent positive **predictive effect** on emotional regulation (β = 0.733, *p* < 0.001) and directly **predict** students' classroom engagement (β = 0.636, *p* < 0.001). Following the introduction of the mediating variable, the positive predictive path of emotional regulation on student engagement remains significant (β = 0.385, *p* < 0.001). The mediation effect test further reveals the **“personality–emotion–engagement” predictive mechanism**. The mediating effect value reaches 0.255, with the confidence interval excluding zero, and an effect contribution rate of approximately 32.1%.

Table 4 presents a detailed account of the structural path coefficients and measurement model loadings among the latent variables through Structural Equation Modeling (SEM), providing core evidence for uncovering the **predictive mechanism** by which teacher personality traits **drive** students' classroom engagement. At the measurement model level, the loadings for the five sub-dimensions of teacher personality traits are all highly significant; notably, Openness (β = 0.919), Conscientiousness (β = 0.939), and Agreeableness (β = 0.967) show particularly prominent performance, which strongly affirms the integrity of the theoretical framework. Concurrently, the three dimensions of classroom engagement and the two emotional regulation strategies also exhibit high standardized loadings (all > 0.820), ensuring the reliability and validity of the latent variable measurements.

**Table 4:** Regression coefficients and measurement model loadings for the structural equation model.

X	→	Y	Non standardized regression coefficient	SE	*z* (CR)	*p*	Standardized regression coefficient
Teacher personality traits	→	Classroom engagement	0.560	0.051	11.085	<0.001	0.636
Teacher personality traits	→	Emotion regulation	0.918	0.078	11.708	<0.001	0.733
Emotion regulation	→	Classroom engagement	0.270	0.038	7.079	<0.001	0.385
Teacher personality traits	→	Openness	0.904	0.041	21.977	<0.001	0.919
Teacher Personality Traits	→	Conscientiousness	0.921	0.038	24.016	<0.001	0.939
Teacher personality traits	→	Agreeableness	0.975	0.035	27.589	<0.001	0.967
Teacher personality traits	→	Extraversion	1.000	–	–	–	0.944
Teacher personality traits	→	Neuroticism	0.628	0.096	6.540	<0.001	0.477
Classroom engagement	→	Emotional engagement	0.859	0.057	15.040	<0.001	0.820
Classroom engagement	→	Cognitive engagement	0.923	0.049	18.776	<0.001	0.894
Classroom engagement	→	Behavioral engagement	1.000	–	–	–	0.933
Emotion regulation	→	Expressive suppression	8.128	0.279	29.106	<0.001	0.972
Emotion regulation	→	Cognitive reappraisal	1.000	–	–	–	0.970

At the structural path level, teacher personality traits exert a potent positive predictive effect on emotional regulation (β = 0.733, *p* < 0.001), and both variables significantly and positively predict student classroom engagement, with path coefficients of 0.636 and 0.385, respectively. These findings clearly demonstrate that teacher personality traits not only directly impact students' classroom performance but also function indirectly through the key mediating variable of emotional regulation. Overall, the *z*-values for all model paths far exceed the critical thresholds, validating the statistical significance and theoretical plausibility of the “trait—emotion—engagement” associative pathways.

Table 5 presents the measurement attributes of the research model, with confirmatory factor analysis (CFA) demonstrating the validity and rigor of latent construct validity. On the independent variable side, teacher personality traits as a higher-order latent variable showed significant standardized loadings (β) across all five dimensions. Notably, openness (0.919), conscientiousness (0.939), and agreeableness (0.967) exhibited exceptionally high construct contributions, effectively addressing concerns about dimension gaps and confirming the high applicability of the Big Five personality framework in this study's sample. On the dependent variable side, the three dimensions of student classroom engagement demonstrated excellent measurement consistency, with loadings exceeding 0.9 (0.927–0.946). This indicates the scale's ability to accurately capture students' emotional, cognitive, and behavioral engagement states. All measurement items showed *z*-values (CR values) well above the critical level with significant *p*-values, further validating the model's convergent validity. Overall, the data reflects the strong representativeness of the measurement indicators used in this study, providing a solid statistical foundation for subsequent path analysis and mediation effect exploration.

**Table 5:** Standardized factor loadings and validity indicators for the measurement model.

Latent variable	Dimensions	β	*z* (CR)	*p*
Teacher personality	Openness	0.919	21.977	<0.001
	Conscientiousness	0.939	24.016	<0.001
	Agreeableness	0.967	27.589	<0.001
	Extraversion	0.944	–	–
	Neuroticism	0.477	6.54	0.001
Classroom engagement	Emotional engagement	0.946	15.11	<0.001
	Cognitive engagement	0.936	14.77	<0.001
	Behavioral engagement	0.927	–	–

Table 6 systematically presents key metrics of Model Fit, with evaluation results consistently demonstrating excellent compatibility between the theoretical model constructed in this study and empirical data. The chi-square freedom ratio (χ^2^/df) of 1.636 is significantly lower than the 3.0 threshold, reflecting an extremely refined model structure with strong explanatory power. The calculated values of root mean square error (RMSEA) and standardized residual root mean square error (SRMR) are 0.064 and 0.052 respectively, both within the excellent range below 0.08, indicating robust performance in handling residual distribution and observed variance with minimal prediction errors. Meanwhile, the comparative fit index (CFI) and non-normalized fit index (TLI), which measure overall model optimization, both exceed the critical threshold of 0.90, reaching 0.94 and 0.93 respectively, further confirming that the improved model surpasses the benchmark model in terms of high-quality empirical research standards. This comprehensive set of fit indicators not only statistically supports the rationality of the path relationship between teacher personality, emotional regulation, and student engagement but also provides a solid model foundation for subsequent analysis of mediating effects. Overall, the data performance is outstanding, fully meeting the rigorous assessment requirements for structural equation modeling in high-level academic journals.

**Table 6:** Overall fit indices for the structural model.

Index	Value	Threshold	Conclusion
χ^2^/df	1.636	<3	Ideal
RMSEA	0.064	<0.08	Good
CFI	0.94	>0.90	Acceptable
TLI	0.93	>0.90	Acceptable
SRMR	0.052	<0.08	Good

Table 7 clearly reveals the structural associations of teacher personality traits with student classroom participation through precise estimation of key path coefficients in the structural model. The data shows that teacher personality traits exhibit a strong and significant positive predictive effect on emotional regulation, with a standardized path coefficient as high as 0.733 (*p* < 0.001) and a critical ratio (*z*-value) of 11.708, fully confirming that individual teacher traits serve as the core antecedent variable driving their emotional regulation strategy selection. At the dependent variable level, emotional regulation significantly promotes student classroom participation (β = 0.385, *p* < 0.001), while the direct effect of teacher personality traits on student participation remains at a high level (β = 0.636, *p* < 0.001). This data strongly supports the theoretical hypothesis of the mediating mechanism in the study. From the perspective of non-standardized coefficients (*B*), each unit increase in teacher personality traits corresponds to corresponding increases of 0.918 units in emotional regulation and 0.560 units in student participation, reflecting robust linear correlations between variables. The significance levels (*p* < 0.001) of all pathways far exceed conventional academic standards, demonstrating that this structural model possesses strong statistical persuasiveness in explaining how teacher personality transforms into students' positive classroom behaviors through the mediating link of emotion. In conclusion, the table results rigorously validate the core logical chain of “trait-emotion-participation” through path value analysis, providing crucial empirical support for understanding interpersonal interaction mechanisms in educational processes.

**Table 7:** Path coefficients and hypothesis testing results of the structural model.

Paths	Non standard coefficient (*B*)	SE	*z* (CR)	*p*	Standardized coefficient (β)
Personality → Emotion regulation	0.918	0.078	11.708	<0.001	0.733
Emotion regulation → Engagement	0.27	0.038	7.079	<0.001	0.385
Personality → Engagement	0.56	0.051	11.085	<0.001	0.636

## 5 Discussion

### 5.1 Main findings

The empirical results provide robust support for the hypothesized associations. Teachers' positive personality traits (Extraversion, Agreeableness, Conscientiousness) were significantly associated with higher student engagement (H1a), and cognitive reappraisal emerged as a key mediating mechanism (H1b, H1c), translating stable traits into a supportive classroom environment (Jusri and Lechner, 2024). The dual-path model (direct effect β = 0.53; indirect effect via emotional regulation = 0.25, accounting for 32.1% of the total effect) highlights reappraisal as a cognitive bridge that fosters instructional resilience, challenging traditional direct-impact models. Consistent with the Big Five framework, Openness showed a strong loading (β = 0.919), suggesting that innovative teachers stimulate students' cognitive curiosity. Neuroticism demonstrated a detrimental but weaker effect (β = 0.477). Teachers prone to emotional instability may default to expressive suppression, creating an emotional disconnect that reduces student involvement. This negative role provides a theoretical counterpoint to the “positive trait” narrative, underscoring the need to include the “dark side” of personality (Ma and Chen, 2024). However, the lower loading of neuroticism may reflect measurement limitations with the NEO-FFI in this teacher sample, or its influence might be indirect and partially buffered by conscientiousness or reappraisal. Future research should examine potential buffers such as organizational support. High factor loadings (e.g., >0.90) indicate strong internal consistency but should be interpreted cautiously, as they may reflect overlapping item content or shared method variance; multi-trait multi-method designs could clarify the unique contribution of each dimension. Beyond confirming expected correlations among extraversion, agreeableness, conscientiousness, cognitive reappraisal, and engagement facets (Sulis and Mercer, 2025; Restoy et al., 2024), the findings suggest a nuanced interplay: extroverted and conscientious teachers naturally elicit trust and structure, but it is their deliberate use of reappraisal that transforms dispositions into engagement gains. This dual-path model positions emotion regulation as an activator aligning dispositional strengths with classroom dynamics. Practical applications include modular training with reappraisal drills, peer-feedback loops, scenario-based workshops, and video analysis to embed emotion management into daily teaching (Schildkamp et al., 2024; Zhi et al., 2024; Tao et al., 2024; Jakonen et al., 2024). Alternative explanations exist: strong associations may partly stem from common method bias or cultural factors (large classes, hierarchical relationships in Chinese higher education), and the mediation pathways may differ in individualistic systems. Cross-cultural studies are needed to test generalizability. Nevertheless, alternative explanations exist. Strong associations among positive traits may partly stem from common method variance or social desirability. The cultural context of Chinese higher education—large class sizes, hierarchical teacher-student relationships, exam orientation—may amplify teacher-centered personality expression while muting student-centered emotional dynamics. Thus, the observed mediation pathways might differ in individualistic or student-centered systems. Cross-cultural comparative studies are needed to test the model's generalizability.

### 5.2 Theoretical and practical implications

While our findings support the mediating role of emotional regulation, it is important to acknowledge that reverse causality cannot be ruled out. Teachers who perceive higher student engagement may be more motivated to regulate their emotions positively, rather than personality traits causing engagement. This alternative direction is equally plausible given the cross-sectional design. Additionally, the relatively weaker relationship for neuroticism warrants attention: Chinese university teachers in our sample reported low mean neuroticism (*M* = 3.40), possibly reflecting a culturally desirable tendency to downplay emotional instability. Thus, the true effect of neuroticism on engagement may be understated. Future research should employ experience sampling or diary methods to capture real-time fluctuations in emotion regulation and engagement, which would provide a more dynamic and less biased picture.

The findings of this study enriched the theoretical framework of research on teachers' personality traits and classroom teaching by further revealing the mediating role of emotional regulation in this relationship. Previous studies primarily focused on the direct effects of teachers' personality traits on student learning outcomes, whereas this study introduced emotional regulation as a mediating variable, providing a more detailed explanatory pathway (Carvalho et al., 2024). This finding not only deepened the understanding of complex psychological mechanisms in teaching contexts but also offered a new perspective for future research in educational psychology.

Practically, the results provided empirical evidence for teacher professional training and classroom management (Huynh, 2024). The findings demonstrated that enhancing teachers' positive personality traits and emotional regulation abilities could directly improve students' classroom engagement and indirectly promote their learning performance through better emotional management (Vo et al., 2025). Specifically, educational institutions could design specialized teacher training programs to help teachers master effective emotional regulation strategies (e.g., cognitive reappraisal) and prioritize the assessment of teachers' personality traits in recruitment and evaluations. Furthermore, encouraging teachers to foster student engagement through positive interactions and emotional support in teaching practice could further optimize teaching outcomes.

### 5.3 Research limitations and future directions

This study has several limitations that warrant careful interpretation. First, the modest sample size (*N* = 154) limits generalizability across Chinese higher education contexts; although *post hoc* power analysis supports medium-effect detection, larger and more diverse samples are needed. Longitudinal designs would also help establish temporal precedence (Zhou et al., 2024). Second, the cross-sectional design precludes causal inference; only associative relationships can be drawn. Third, common method and perceptual bias are concerns because all variables were teacher-reported; Harman's test alone cannot rule out inflation due to response style or social desirability. Multi-source data (e.g., student reports, observations) are recommended. Fourth, convenience sampling via an online platform may have introduced self-selection bias; the sample overrepresented female teachers (62.3%) and lecturers (74.0%), limiting generalizability to male or senior faculty. Probability sampling is needed. Fifth, other mediators (teaching styles, classroom climate) might also play a role (Deng, 2024).

Measurement-related issues also deserve attention. The classroom engagement scale was originally developed for mathematics contexts, and despite pilot testing, nuances of non-mathematics disciplines may not be fully captured. The ERQ was designed for general populations and may not fully address teacher-specific emotion regulation (e.g., managing classroom disruptions). Additionally, several bivariate correlations and factor loadings exceeded 0.90 (e.g., Extraversion–Agreeableness, *r* = 0.938). Although HTMT and VIF values did not indicate discriminant validity violations or problematic collinearity, such strong associations suggest shared variance among the Big Five dimensions; they should not be interpreted as orthogonal. Culturally, Chinese teachers' emphasis on emotional restraint and role-based authority may shape personality expression and emotion regulation differently than in Western contexts. Thus, the proposed associative model requires cross-cultural replication.

In summary, the study reveals complex associations among teacher personality, emotional regulation, and student engagement, while providing clear directions for future research.

## 6 Conclusion

This study utilized Structural Equation Modeling (SEM) to examine and validate the underlying mechanism of “personality traits–emotion regulation–classroom engagement” among Chinese university teachers. The findings indicate that teacher personality, as a holistic construct, exerts a significant and positive direct predictive effect on students' classroom engagement (β = 0.636$), with the high factor loadings of Openness, Conscientiousness, and Agreeableness underscoring the core value of positive psychological traits in driving an interactive instructional climate. Further path analysis reveals that emotion regulation—specifically the cognitive reappraisal strategy—plays a pivotal mediating role as a “bridge” in the trait-engagement association; the indirect effect (0.255) accounts for 32.1% of the total effect, providing robust evidence that stable personality advantages must be translated through dynamic emotional management strategies to effectively elicit active student participation. Consequently, higher education institutions should integrate emotion management training into professional development frameworks, utilizing specialized cognitive reappraisal drills to enhance teachers' psychological resilience in complex environments, thereby optimizing teacher-student interactions and scientifically improving instructional quality while providing critical theoretical support for evidence-based practice in higher education.

## Data Availability

The original contributions presented in the study are included in the article/supplementary material, further inquiries can be directed to the corresponding author/s.
